# Risk Communication Concerning Welding Fumes for the Primary Preventive Care of Welding Apprentices in Southern Brazil

**DOI:** 10.3390/ijerph120100986

**Published:** 2015-01-19

**Authors:** Marta Regina Cezar-Vaz, Clarice Alves Bonow, Joana Cezar Vaz

**Affiliations:** 1 School of Nursing, Federal University of Rio Grande, Rio Grande, RS 96203-900, Brazil; 2 Graduate Program on Nursing, Federal University of Pampa, Uruguaiana, RS 97500-970, Brazil; E-Mail: claricebonow@unipampa.edu.br; 3 School of Chemistry and Food, Federal University of Rio Grande, RS 96202-900, Brazil; E-Mail: joanacezarvaz@yahoo.com.br

**Keywords:** risk communication, welding trainees, primary prevention

## Abstract

This study’s aim was to assess the perceptions of welding apprentices concerning welding fumes being associated with respiratory and cardiovascular disorders and assess the implementation of risk communication as a primary prevention tool in the welding training process. This quasi-experimental, non-randomized study with before-and-after design was conducted with 84 welding apprentices in Southern Brazil. Poisson Regression analysis was used. Relative Risk was the measure used with a 95% confidence interval and 5% (*p* ≤ 0.05) significance level. Significant association was found between perceptions of worsened symptoms of respiratory disorders caused by welding fumes and educational level (*p* = 0.049), the use of goggles to protect against ultraviolet rays (*p* = 0.023), and access to services in private health facilities without insurance coverage (*p* = 0.001). Apprentices younger than 25 years old were 4.9 times more likely to perceive worsened cardiovascular symptoms caused by welding fumes after risk communication (RR = 4.91; CI 95%: 1.09 to 22.2). The conclusion is that risk communication as a primary preventive measure in continuing education processes implemented among apprentices, who are future welders, was efficacious. Thus, this study confirms that risk communication can be implemented as a primary prevention tool in welding apprenticeships.

## 1. Introduction

Primary preventive actions are developed and organized to avoid or minimize risks posed to the heath of individuals or a collective. Preventive measures somewhat express priorities established and directed by public policies [1]. In the specific case presented here, primary prevention focuses on reducing diseases caused by the exposure of welders and future workers to harmful compounds. In this context, the primary preventive measure we propose to enhance the well-being of these workers and future workers is risk communication [2,3,4,5,6,7,8,9,10].

Risk communication refers to an interactive process in which information and opinions are exchanged among interested parties, and include messages concerning events that pose risks to workers and how to identify, analyze and manage such risks [2,10]. For risk communication to be effective, the characteristics of the event posing a risk to workers need to be identified because these characteristics influence the individuals’ perceptions of risks.

Thus, risk communication, along with public participation and conflict resolution, encourages the modification of individual and collective behavior in the face of events that pose risks to health [2]. The use of risk communication as a primary prevention tool is found in different working environments, such as among farmers [11], chemical industries [12], and technological education schools [13], among others.

This study’s aim was to apply the communication of risks in welding apprenticeship programs. The Laboratory of Socio-Environmental Process Studies and Collective Health Promotion (LAMSA) research group has developed research in this context in the Socio-Environmental Laboratory of Occupational Health at the School of Nursing, Federal University of Rio Grande [13,14]. The perceptions of welding apprentices concerning welding fumes as associated with respiratory and cardiovascular disorders was used to classify events that pose risks to health and that can be communicated in the apprenticeship process. The reason is that the awareness of apprentices concerning this event can be strengthened or even modified during the training process to ensure safer occupational practices.

Welding is a common industrial process but one with the potential to be extremely harmful, and detrimental to the health of welders [15]. One of the main risk factors to which welders are exposed is welding fumes and gases. The main disorders caused by such exposure include respiratory disorders as reported in studies conducted in Denmark [16], Iran [17] and Malaysia [18], and cardiovascular disorders as reported by studies conducted in the United States [19] and Denmark [20]. There are, however, few studies addressing welding apprenticeship programs [21,22] and the implementation of risk communication as a primary prevention tool to advise future workers concerning the exposure of welding fumes being associated with respiratory and cardiovascular disorders.

Even though evidence provided in the literature concerning the association between exposure to welding fumes and cardiovascular disorders is somewhat weak and inconclusive, there are studies showing that both respiratory and cardiovascular disorders may accrue from exposure to fumes due to the metals released during the process. Metals are released due to the type of welding processes performed. The metal arc used by the apprentices in the welding process produces heat from electrodes for bonding metals. In this type of welding process, heat coming from the opening of the electric arc is used to melt the electrodes and the flux, which has a gaseous discharge upon melting that encases the electrodes. All welding processes produce fumes and gases, but even more fumes are produced during manual arc welding. Welding fumes are vapors that originate from metals and electrodes’ metallic compounds that react with air and then condense, forming particles [15,23,24,25]. At least 13 metals, such as cobalt, beryllium, molybdenum, cadmium, chrome, copper, iron, manganese, nickel, lead, antimony, vanadium and zinc [25,26] are produced during the welding process.

Such characteristics reinforce the need to communicate risks to apprentices, who are workers without experience. Even experienced workers and those in continuing education programs, benefit from risk communication. Even though this study’s objectives do not include clinical studies or chemical analyses, the study is based on clinical and chemical evidence and is designed to identify the perceptions of apprentices concerning associations between welding fumes and respiratory and cardiovascular disorders.

Therefore, risk communication is seen as a primary preventive measure intended to reduce exposure of individuals to welding fumes in their future working place; *i.e.*, apprentices are sensitized to self-care and can also become role models to their future co-workers.

One of the roles of public health agents in the occupational sphere is to invest in the process of health education to improve workers’ self-care within their work environments. Hence, primary health care (PHC) providers can use risk communication as an efficient primary prevention tool.

Given the previous discussion, this study’s aim was to analyze the perceptions of welding apprentices concerning welding fumes as being associated with respiratory and cardiovascular disorders and to assess the role of risk communication as a primary prevention tool in a welding apprenticeship program.

## 2. Methods

This is a quasi-experimental, non-randomized study with a before-and-after design. The sample comprised 84 welding apprentices attending a private educational institution in Southern Brazil, the objective of which is to develop and improve the Brazilian shipbuilding industry. The 84 apprentices composed six classes, each with approximately 14 individuals. Even though this is not a very large sample, there is a need to investigate welding apprentices because this population is usually addressed only in technical studies [27,28] focusing on the efficiency of welding processes. Hence, encouraging and conducting studies with welding apprentices, even if in small samples, deepens knowledge concerning the health of these future workers and contributes to the accumulation and reproduction of data for occupational health surveillance [1] in both working and apprenticeship environments.

This teaching facility qualifies professionals and promotes the improvement of industry products and processes by providing technical and technological teaching programs and services. The welding training program has a duration of four months and consists of theoretical and practical classes. The criterion used to select the participants was being at the beginning of the practical classes, regardless of previous experience. This criterion is justified because both inexperienced and experienced individuals compose the group; welding practice requires that even workers with prior experience update their knowledge concerning different types of processes [29,30]. Because the group gathers inexperienced and experienced individuals in the same program, the implementation of risk communication in the teaching process is improved as a primary prevention tool.

The literature search was based on the guiding question: “How does one communicate the risks associated with respiratory and cardiovascular disorders to welding apprentices?” We identified studies addressing perception and risk communication [2,3,4,5,6,7,8,9,10], clinical trials addressing respiratory [15,16,17,18,31,32] and cardiovascular disorders [19,20,33,34,35,36], and the chemical components of welding fumes [23,24,26], and studies addressing preventive measures implemented among welding apprentices [21,22]. This review enabled the construction and development of a risk communication strategy regarding respiratory and cardiovascular disorders that is directed to welding apprentices. In addition to this search in the literature, the researchers participated in theoretical welding classes, the knowledge of which was implemented in the risk communication strategy. This participation was important for the researchers to understand the activity of welding because risk communication requires clear understanding be established between those transmitting the message and those receiving it [10]. Therefore, participation in welding theory classes improved risk communication concerning the development of respiratory and cardiovascular disorders. Note that risk communication in the training process is a measure to prevent respiratory and cardiovascular disorders. Therefore, effective communication concerning occupational risks within a training program requires the ability to convince someone (the apprentice) and the establishment of a trust relationship between students and communicators (in this case, LAMSA researchers).

A pre-test was initially applied, *i.e.*, before content concerning welding fumes being a chemical risk with the potential to cause respiratory and cardiovascular disorders was presented. The questionnaire was composed of two parts. The first addressed the participants’ characteristics (age, sex, education, ethnicity and marital status); whether the participants had prior experience with welding; daily exposure to welding fumes and gases; smoking; access to health services; and the use of personal protective equipment (PPE). The second part presented 18 variables related to the identification of respiratory disorders (symptoms of asthma or bronchitis such as dyspnea, cyanosis, or rhinitis), cardiovascular disorders (symptoms of cardiovascular disorders—hypertension associated with tachycardia, hypotension, or edemas in lower limbs), and the use of PPE during the welding process. All variables were measured on a 5-point Likert scale ranging from 0 (never felt/used) to 4 (always felt/used). The pre-test questionnaire was applied to the participants who had already attended theoretical classes and had begun practical classes, *i.e.*, in the second half of the program, or the last four months.

Content (message) concerning welding fumes presenting a chemical risk potentially causing respiratory and cardiovascular disorders was transmitted immediately after the pre-test questionnaire. The LAMSA participants explained to the welding apprentices that exposure to chemical compounds released in welding fumes (cobalt, beryllium, molybdenum, cadmium, chrome, copper, iron, manganese, nickel, lead, antimony, vanadium and zinc) [26] potentially lead to respiratory disorders (asthma, cyanosis [21,37,38], bronchitis, dyspnea [31] and rhinitis [32]) and cardiovascular disorders (tachycardia, bradycardia [33,34], hypertension [19], hypotension [39] and edema in the lower limbs [40]), according to evidence reported concerning the specificity of each component. Dialogues were used to expose the content so that the participants had a chance to clarify doubts and share their experiences concerning the topic under discussion—welding fumes as chemical risks causing respiratory and cardiovascular disorders.

The post-test questionnaire was applied after two months of administering content concerning welding fumes as a chemical risk causing respiratory and cardiovascular disorders. The questionnaire addressed the same questions contained in the second part, as previously mentioned; the participants had already completed the first part (characterization). Hence, risk communication was implemented at three different points in time in each of the six classes, totaling 18 meetings (Table 1).

**Table 1:** Scheme of the risk communication process implemented among welding apprentices.

Risk Communication Process	
Literature review	Papers addressing risk perception and communication [2,3,4,5,6,7,8,9,10], respiratory [15,16,17,18,31,32] and cardiovascular [19,20,33,34,35,36] disorders, chemical compounds of welding fumes [23,24,26], and prevention among welding apprentices [21,22].
Attending theoretical classes	The content administered during classes included: types of wires; gases used during welding; occupational safety; and technical design, among other content.
Planning of risk communication	The planning for the meetings and topics to be discussed during risk communication were based on knowledge acquired from the literature and in theoretical classes.
Application of pre-test	The questionnaire was applied during a theoretical class.
Implementation of risk communication	Dialogues were used to transmit content to the apprentices regarding exposure to chemical risks, including illustrations presenting risks and preventive measures.
Application of post-test	Post-test was applied two months after risk communication was implemented.
Prospects for future cooperation between the welding apprenticeship institution and LAMSA research group.	Discussion concerning potential research topics to identify and communicate other risks and preventive measures.

Descriptive analysis was used for the variables of age, experience, and daily exposure, which were presented in terms of mean and standard deviation or median and interquartile range, according to the distribution of variables. Because the mean is influenced by extreme values in asymmetric distributions, the median was the measure of central tendency chosen to summarize data in this type of distribution. The categorical variables of sex, education, ethnicity, marital status, smoking, access to health services, and the use of PPE, were described using absolute and relative frequencies.

To compare the means between the before-and-after groups (the group that perceived worsened symptoms of cardiovascular and/or respiratory disorders and the group that did not present such a perception), Student’s t-test was used for independent samples and the Mann-Whitney test was used in cases of asymmetry. Pearson’s Chi-square test or Fisher’s exact test were used to compare proportions.

In the multivariate analysis, dichotomous outcomes were created to better understand the meanings of the perceptions of welding apprentices concerning welding fumes causing respiratory and cardiovascular disorders, before and after risk communication was implemented. The outcomes were categorized as: value one (1) presented worsened symptoms and, therefore, acquired a stronger awareness that symptomatology may be associated with welding fumes; value zero (0) there were no changes or no improvement was noticed after risk communication, therefore, awareness that symptomatology may be associated with welding fumes did not improve.

Poisson Regression analysis was used to control for confounding factors. The criterion to add variables in the model was that it presented a *p* < 0.20 value in the bivariate analysis. The measure used was Relative Risk (RR) with a 95% confidence interval and significance level at 5% (*p* ≤ 0.05). The analysis was conducted in the SPSS program, version 21.0.

The study was conducted in accordance with the Declaration of Helsinki, all individuals consented and signed free and informed consent forms, and the Institutional Review Board at Federal University of Rio Grande approved the project (109/2010).

## 3. Results

### 3.1. Descriptive Analysis

The sample comprised 84 welding apprentices aged 27.5 years old (±7.2 years) on average. Most were male (78.6%), Caucasian (55.4%), single (57.3%), and had completed high school (54.2%), while 14 (16.7%) individuals reported smoking. A total of 32 (38.1%) out of 84 individuals reported prior experience of 12 months (median) with welding, with a minimum of two months and a maximum of 120 months (10 years); the average daily exposure was 5.5 hours (±2.2). Table 2 presents the sample characterization.

**Table 2:** Sample characterization.

Variable	n = 84
Age (years)—media ± SD *	27.5 ± 7.2
Gender—n(%)	
Male	66 (78.6)
Female	18 (21.4)
Age range—n(%)	
<25 years	32 (40.5)
25 to 29 years	20 (25.3)
≥30 years	27 (34.2)
Ethnicity—n (%)	
Caucasian	46 (55.4)
African descendant	21 (25.3)
Mixed	15 (18.1)
Indigenous	1 (1.2)
Marital status—n (%)	
Single	47 (57.3)
Married	33 (40.2)
Separated	2 (2.4)
Education—n(%)	
Middle school, incomplete	11 (13.3)
Middle school	3 (3.6)
High school, incomplete	15 (18.1)
High school	45 (54.2)
Undergraduate education, incomplete	5 (6.0)
Bachelor’s degree	3 (3.6)
Graduate education	1 (1.2)
Welding experience—n(%)	
Yes	32 (38.1)
No	52 (61.9)
Welding experience (months)—md **^†^** (P25–P75)	12 (5–23)
Daily exposure to welding fumes (hours)—mean ± SD	5.5 ± 2.2
Smoking—n (%)	14 (16.7)
Access to health services through **—n (%)	
PHC services	25 (29.8)
Public Emergency rooms	37 (44.6)
Health insurance	43 (51.2)
Private health services (with no health insurance coverage)	7 (8.4)

Notes: * Standard deviation; **^†^** median; P25 = percentile 25; P75 = percentile 75; ** multiple response question.

Most participants reported access to emergency rooms in the Brazilian public healthcare system (44.6%) and through healthcare insurance/plans (51.2%). Almost the entire sample (98.8%) used PPE during the training program: gloves, apron, safety glasses, welding masks, and earplugs. No significant differences (*p* > 0.05) were found in the comparison between reports of respiratory and cardiovascular symptoms before and after risk communication (Table 3), meaning that the perception that symptoms may be associated with the practice of welding did not show significant statistical change in frequency.

**Table 3:** Comparison of outcomes before and after the risk communication.

Variable	Before (n = 84) n (%)	After (n = 76) n (%)	*p* *
*Respiratory disorders*			
Dyspnea			0.091
Never	60 (72.3)	52 (68.4)	
Almost never	9 (10.8)	7 (9.2)	
Sometimes	14 (16.9)	15 (19.7)	
Often	0 (0.0)	2 (2.6)	
Cyanosis			0.083
Never	81 (98.8)	72 (94.7)	
Almost never	1 (1.2)	4 (5.3)	
Rhinitis			0.222
Never	71 (86.6)	62 (80.5)	
Almost never	5 (6.1)	8 (10.4)	
Sometimes	5 (6.1)	6 (7.8)	
Often	0 (0.0)	1 (1.3)	
Always	1 (1.2)	0 (0.0)	
*Cardiovascular disorders*			
Edema in the lower limbs			0.157
Never	81 (96.4)	72 (94.7)	
Almost never	3 (3.6)	2 (2.6)	
Sometimes	0 (0.0)	1 (1.3)	
Often	0 (0.0)	1 (1.3)	
Always	0 (0.0)	0 (0.0)	
Hypertension			0.655
Never	79 (94.0)	71 (93.4)	
Almost never	2 (2.4)	2 (2.6)	
Sometimes	1 (1.2)	3 (3.9)	
Often	1 (1.2)	0 (0.0)	
Always	1 (1.2)	0 (0.0)	
Hypotension			0.129
Never	75 (89.3)	66 (86.8)	
Almost never	6 (7.1)	6 (7.9)	
Sometimes	3 (3.6)	4 (5.3)	

Notes: * Wilcoxon Test.

No significant differences were found in regard to the use of PPE before and after risk communication. This result is directly linked to the nature of the process under study; *i.e.*, the participants were attending practical classes, which reinforces the prevalence of PPE.

### 3.2. Multivariate Analysis

Even though the results were not significant, dichotomous outcomes were created to facilitate understanding concerning the meaning of the apprentices’ perceptions regarding welding fumes causing respiratory and cardiovascular disorders, before and after risk communication. The outcomes were categorized as: value one (1) reported worsened symptoms, thus acquired a more accurate perception that symptoms may be associated with welding fumes; value zero (0) no changes were perceived or symptoms were perceived to have improved after risk communication, thus perceptions that symptoms may be associated with welding fumes did not improve. After establishing the dichotomous outcomes, they were associated with the study’s variables. These associations are presented in Table 4.

**Table 4:** Variables associated with worsened respiratory and cardiovascular symptoms.

Variable	Perception of Worsened Self-Reported Respiratory Symptoms (n = 14; 18.4%)	No Perception of Worsened Self-Reported Respiratory Symptoms (n = 62; 81.6%)	p	Perception of Worsened Self-Reported Cardiovascular Symptoms (n = 8; 10.5%)	No Perception of Worsened Self-Reported Cardiovascular Symptoms (n = 68; 89.5%)	*p*
Gender			0.476 *			0.672 **^†^**
Male	10 (71.4)	50 (80.6)		6 (75.0)	54 (79.4)	
Female	4 (28.6)	12 (19.4)		2 (25.0)	14 (20.6)	
Age			0.313 *			0.095 *
<25 years old	8 (61.5)	24 (40.0)		6 (75.0)	26 (40.0)	
25 to 29 years old	3 (23.1)	16 (26.7)		2 (25.0)	17 (26.2)	
≥30 years old	2 (15.4)	20 (33.3)		0 (0.0)	22 (33.8)	
Ethnicity			0.167 *			0.988 **^†^**
Caucasian	5 (38.5)	37 (59.7)		5 (62.5)	37 (55.2)	
African descendant	6 (46.2)	11 (17.7)		3 (37.5)	30 (44.8)	
Mixed	2 (15.4)	13 (21.0)				0.533 *
Indigenous	0 (0.0)	1 (1.6)		6 (75.0)	36 (54.5)	
Marital status			0.852 *	2 (25.0)	29 (43.9)	
Single	8 (61.5)	34 (55.7)		0 (0.0)	1 (1.5)	
Married	5 (38.5)	26 (42.6)				0.711 **^†^**
Separated	0 (0.0)	1 (1.6)		6 (75.0)	44 (65.7)	
Education			0.049 *	2 (25.0)	23 (34.3)	
High school or undergraduate studies	12 (92.3)	38 (61.3)				0.472 **^†^**
Incomplete high school	1 (7.7)	24 (38.7)		4 (50.0)	25 (36.8)	
Welding experience			0.481 *	4 (50.0)	43 (63.2)	
Yes	7 (50.0)	22 (35.5)		12.5 (12–21)	7 (4-18)	0.310 **
No	7 (50.0)	40 (64.5)		6.75 ± 1.83	5.33 ± 2.26	0.092 ***
Welding experience (months)	13 (5–18)	9.5 (4–19.5)	0.500 **	0 (0.0)	12 (17.6)	0.342 **^†^**
Daily exposure to welding fumes (hours)	4.8 ± 2.1	5.6 ± 2.3	0.228 ***			
Smoking	1 (7.1)	11 (17.7)	0.447 *	2 (25.0)	22 (32.4)	1.000 **^†^**
Access to health services				3 (37.5)	18 (26.9)	0.679 **^†^**
PHC services	3 (21.4)	21 (33.9)	0.528 *	2 (25.0)	20 (29.4)	1.000 **^†^**
Public emergency rooms	5 (38.5)	16 (25.8)	0.497 *	0 (0.0)	7 (10.4)	1.000 **^†^**
Private health insurance	7 (50.0)	15 (24.2)	0.099 *	6 (75.0)	54 (79.4)	0.672 **^†^**
Private health services (with no health insurance coverage)	3 (23.1)	4 (6.5)	0.095 ^†^	2 (25.0)	14 (20.6)	

Notes: * Chi-square test; ** Mann-Whitney test; *** Student’s t test; **^†^** Fisher’s exact test.

There was significant association between perceptions of worsened self-reported symptoms of respiratory disorders caused by welding fumes and educational level (*p* = 0.049). Apprentices perceiving worsened symptoms presented higher educational levels (high school or undergraduate studies) when compared to those who did not present such perceptions (92.3% *vs.* 61.3%).

Significant association was found in regard to the use of PPE. Apprentices perceiving worsened symptoms of respiratory disorders also presented less frequent use of safety glasses to protect against ultraviolet rays when compared to those who did not present such perceptions (15.4% *vs.* 54.8%; *p* = 0.022). Associations with the outcome were not significant (*p* > 0.05) in regard to the use of PPE before and after risk communication. In order to control confounding factors, variables with *p* < 0.20 in the bivariate analysis were included in a multivariate Poisson regression model, as shown in Table 5 and Table 6.

**Table 5:** Multivariate Poisson Regression analysis assessing factors independently associated with the perception of worsened respiratory symptoms.

Variables	RR (CI 95%)	*p*
Use of safety glasses to protect against ultraviolet rays	0.21 (0.06 to 0.81)	0.023
Access to private health services using health insurance	1.93 (0.76 to 4.90)	0.167
Access to private health services without insurance coverage	3.84 (1.75 to 8.39)	0.001
High school or undergraduate studies	5.48 (0.74 to 40.8)	0.097

Notes: RR = Relative Risk; CI 95%: Confidence Interval.

**Table 6:** Multivariate Poisson Regression analysis assessing factors independently associated with perceptions of worsened cardiovascular symptoms.

Variables	RR (CI 95%)	*p*
Age < 25 years old	4.91 (1.09 to 22.2)	0.039
Access to public healthcare services (university emergency room)	4.43 (1.35 to 14.6)	0.014
Time of daily exposure (h)	1.18 (0.85 to 1.63)	0.322

Notes: RR = Relative Risk; CI 95%: Confidence Interval.

After adjusting for ethnicity, education and income, the following remained statistically associated with the perception that self-reported respiratory symptoms worsened due to exposure to welding fumes: the use of safety glasses to protect against ultraviolet rays (*p* = 0.023) and access to private healthcare services without insurance coverage (*p* = 0.001). The apprentices using protective glasses during practical classes were 79% less likely to perceive worsened respiratory symptoms after risk communication (RR = 0.21; CI 95%: 0.06 to 0.81). Individuals with access to private healthcare services but who did not have health insurance were 3.84 times more likely to perceive worsened respiratory symptoms caused by welding fumes after risk communication compared to those who accessed healthcare services by other means (RR = 3.84; CI 95%: 1.75 to 8.39). Having completed high school or attended undergraduate studies was nearly significant (RR = 5.48; CI 95%; 0.74 to 40.8; *p* = 0.097).

No significant association was found between perceptions of worsened self-reported cardiovascular symptoms caused by welding fumes and any of the study’s variables (*p* > 0.05), as shown in Table 4. Additionally, associations were not significant between perceptions of worsened symptoms and the use of PPE (*p* > 0.05).

Even though no significant association was found, the variables with *p* < 0.20 in the bivariate analysis were included in a multivariate Poisson Regression model. After adjustment, time of exposure was associated with the perception that self-reported cardiovascular symptoms caused by welding fumes worsened; with age (*p* = 0.039); and with access to a public emergency room (*p* = 0.014).

Apprentices younger than 25 years old were 4.9 times more likely to perceive worsened cardiovascular symptoms after risk communication (RR = 4.91; CI 95%: 1.09 to 22.2). Those with access to public healthcare services though emergency rooms were 4.43 times more likely to perceive worsened cardiovascular symptoms when compared to apprentices who accessed healthcare services by other means (RR = 4.43; CI 95%: 1.35 to 14.6).

## 4. Discussion

This study’s findings reveal that the perception that exposure to welding fumes triggers respiratory and cardiovascular disorders is stronger for apprentices with higher education levels. This is also reported by one study addressing other types of workers, in which a low level of knowledge concerning occupational risks may be associated with low levels of education [41]. This evidence corroborates the application of risk communication as a primary prevention tool in apprenticeship programs like the one presented in this study.

The implementation of primary prevention in training programs represents a possibility to improve the knowledge of apprentices regarding occupational safety and encourages self-care. Another aspect is that the apprentices’ mindful behavior may positively affect the behavior of future co-workers. Hence, welding workers need to constantly improve and update their knowledge, considering the deleterious effects of this occupation on health. These effects directly impact the bodies of workers and are caused by exposure to compounds presented in welding fumes and gases and in suspension in the working environment, as evidence shows [24].

The use of PPE was not statistically significant as a primary preventive measure before or after risk communication. The use of PPE is acknowledged worldwide as an efficacious measure against the inhalation of welding fumes and gases, minimizing harmful effects on workers’ bodies. According to ESAB, a world leader in the manufacturing of welding and cutting equipment, the most frequently used PPE items are welding masks, respiration systems, safety glasses, protective welding gloves, leather jackets, sleeves and hoods, and boots [42].

The exception in this study was the use of safety glasses to protect against ultraviolet radiation. The infrequent use of this item was associated with a clearer perception that welding fumes lead to respiratory disorders. This study was conducted in a teaching environment and for this reason PPE is a requirement for apprentices to attend practical classes; however, the institution does not provide this specific item. The need for safety glasses was reinforced during risk communication and the non-use of safety glasses led apprentices to identify respiratory disorders as being triggered by welding fumes and also to reflect upon the need for this protective item. Therefore, knowledge regarding the use of PPE as a primary preventive measure determines the behavior of apprentices and their raised awareness regarding the use of safety glasses is an important marker of the risk communication strategy implemented in this study.

Risk communication promotes greater sensitization of apprentices regarding the use of PPE as a primary preventive measure. Different studies present controversial results regarding the use of PPE. One study verifying the awareness of 330 Nigerian welders concerning risks posed to health as related to and their adherence to occupational safety rules reports that awareness of both occupational risks and adherence to protective measures was high [43]. Comparing Brazil, where this study was conducted, with Nigeria may be controversial due to the level of development of both countries. Nonetheless, even though Nigeria is an underdeveloped country, the working conditions of Nigerian welders are similar to the working conditions of these workers in Brazil, which is a developing country.

Another study, conducted with 271 Nigerian welders, reported that 37 (13.7%) individuals did not use safety glasses [44]. One intervention study addressing 285 Nigerian welders reports low awareness on the part of welders in regard to the use of PPE, both in the intervention group and the control group, except for the use of safety glasses and gloves [45].

Even studies that do not find evidence that using the entire set of equipment is a guarantee of full protection should encourage the use of PPE as a primary preventive measure, as in the case of a specific study that aimed to determine efficacy of filters used as respiratory protection during welding to reduce inhalation of particles and gases. The study reports that the four devices under study (surgical masks, cotton masks, activated charcoal masks, and N95 respirator masks) were efficient in blocking the inhalation of particles but were not efficient in blocking the gases found in welding fumes [25]. These are the respiratory protective devices most frequently used by welders and other workers exposed to chemical compounds, such as painters and plate makers. Surgical and cotton masks are the simplest found in the market. They are made with cotton fiber and are usually indicated for protecting against biological risks such as the H1N1 virus [46]. Activated charcoal masks are efficient against chemicals such as pesticides used by farmers [47]. These masks have internal filters of activated charcoal, which protect against vapors and suspended particles. The N95 respirator is usually used in industries and hospitals to protect against welding fumes and Mycobacterium tuberculosis. It is more efficient than the others against airborne particles when it is tightly sealed [48].

Evidence highlights controversies regarding the use of PPE as an occupational primary preventive measure, however studies reinforce the assertion that protective equipment should be encouraged and reinforced during the training process. This was the purpose of this study, by implementing a risk communication strategy among welding apprentices.

In addition to increasing their concern with personal protection equipment, apprentices also should become concerned with their lifestyle and habits, such as smoking. Most of this study’s participants were non-smokers; however, 14 (16.7%) reported being smokers. Association between smoking and exposure to welding fumes exacerbates harms caused to the respiratory tract. One follow-up conducted in Austria to reduce the risk of respiratory disorders associated with welding highly recommends welders quit smoking [49].

Another piece of evidence revealed in this study is the more common perception by apprentices that welding fumes lead to respiratory and cardiovascular disorders. The relationship between access to health services and the identification of this perception constitutes a secondary preventive measure for respiratory and cardiovascular symptoms caused by welding fumes. This study’s evidence shows that having access to healthcare services promotes greater sensitization to understanding that welding fumes can cause respiratory and cardiovascular disorders. Even though it was not the objective of this study, it is important to note that both primary and secondary healthcare services, private and public PHC units and outpatient clinics, should be prepared to recognize welding-related respiratory and/or cardiovascular problems [50]. Seeking the guidance of PHC services was emphasized in the implementation of risk communication as a primary preventive measure [51].

Another aspect to highlight is the apprentices’ perceptions that welding fumes cause cardiovascular disorders. This perception was more frequently observed in the implementation of risk communication due to the duration of these disorders, indicating that the risk communication strategy was effective for those younger than 25 years old; *i.e.*, the communication resulted in an increased perception that cardiovascular disorders are associated with welding. We emphasize the need to implement primary prevention in regard to respiratory and cardiovascular disorders caused by welding fumes among apprentices, because these individuals have a long productive period and preventive measures represent a positive qualifier for the future lives of these workers.

Risk communication is an important preventive measure to sensitizing welding apprentices in regard to the harmful effects of welding fumes that lead to respiratory and cardiovascular disorders, as evidence showing that welding fumes are associated with these disorders affirms. One relevant aspect is that future welders, especially those in the shipbuilding industry, will be exposed to chemical compounds such as manganese [52], and aluminum [53], among other substances. According to the Occupational Safety and Health Administration (OSHA) [26], manganese, a compound produced in welding fumes, is linked to respiratory disorders; it impairs respiratory function [54,55] and causes cardiovascular disorders due to hypotension [39]. Even though aluminum is not mentioned by OSHA [26] as a compound related to welding fumes, it is seen as a component possibly linked to occupational asthma [37], decreased expiratory and cardiovascular flow [56] due to cardiac arrhythmias [34].

This study does not present a chemical analysis of the compounds present in welding fumes; however, both the literature and empirical evidence of the local shipbuilding industry show these chemicals to be present in welding fumes [1].

## 5. Conclusions

The conclusion reached in this study is that risk communication was an efficacious primary preventive measure implemented in the training process of future welders. This study produced evidence based on the sensitization of apprentices concerning the perception that welding fumes cause respiratory and cardiovascular disorders, a perception especially found among those with higher educational levels and younger than 25 years old. Those with access to public and private healthcare services also experienced increased sensitization, showing the need for these services to be prepared to provide guidance to these individuals. This shows the need for future studies to address this subject in healthcare services. Additionally, vaccination against pneumonia should be considered as a preventive measure among these individuals, since they become more susceptible to respiratory problems due to the welding activity in which they are engaged.

Even though the risk communication strategy was implemented in a specific group of apprentices to raise their level of awareness regarding the risk of welding fumes to cause respiratory and cardiovascular disorders, this study contributes to improved risk communication as a preventive measure among individuals receiving training. Hence, risk communication is confirmed as a primary preventive measure in the welding apprenticeship process. This study also encourages those interested in the development of risk communication among groups receiving training and is as a useful source of data for occupational health surveillance.
